# Study on the reproducibility of the Frankfort horizontal plane based on CBCT 3D reconstruction and comparative analysis with lateral radiographs

**DOI:** 10.3389/fsurg.2026.1786145

**Published:** 2026-07-16

**Authors:** Zhiwen Li, Liuli Jin, Xueyang Zhang, Pan Jiang, Yuan Lin, Qun Yang

**Affiliations:** 1 Department of Stomatology, The Eighth Affiliated Hospital of Southern Medical University (The First People’s Hospital of Shunde), Foshan, China; 2 Stomatological Hospital, School of Stomatology, Southern Medical University, Guangzhou, China

**Keywords:** 3D reconstruction, CBCT, facial deformity, Frankfort horizontal plane, lateral radiograph, reproducibility

## Abstract

**Objective:**

To investigate the reproducibility of the Frankfort horizontal plane (FHP) established using CBCT-based three-dimensional reconstruction and to evaluate reproducibility errors generated by CBCT and lateral cephalometric reference-construction workflows in patients with facial deformities.

**Methods:**

A total of 27 patients with facial deformities were enrolled. All subjects underwent both CBCT scanning and lateral cephalometric radiography. For the CBCT data, three-dimensional craniofacial models were reconstructed using Mimics 21.0 and Geomagic Studio 2014, and the FHP was established accordingly. For lateral cephalometric radiographs, bilateral orbitale and porion landmarks were manually identified using Adobe Photoshop 2024. Two experienced orthodontists independently performed landmark identification twice. Landmark positional errors and plane angular errors were calculated. Intra-class correlation coefficients (ICCs) were used to assess reproducibility, Bland–Altman analysis was applied to evaluate agreement, and paired t tests were performed to evaluate differences in reproducibility errors generated by the CBCT and lateral cephalometric workflows.

**Results:**

The CBCT 3D reconstruction method demonstrated good reproducibility for all landmarks and planes when establishing FHP, while lateral cephalometric measurements exhibited larger reproducibility errors than the CBCT workflow (*p* < 0.05).

**Conclusion:**

CBCT-based three-dimensional reconstruction demonstrated high reproducibility for Frankfort horizontal plane construction. This technique may provide a reliable reference framework for the diagnosis and treatment planning of patients with facial deformities.

## 1 Introduction

The Frankfort horizontal plane (FHP) is a fundamental reference plane in craniofacial anthropometry and is widely applied in orthognathic surgery, orthodontic treatment, and the assessment of facial deformities (1). As a stable spatial reference, the FHP plays an indispensable role not only in cephalometric analysis but also in facial symmetry evaluation, deformity diagnosis, surgical planning, and postoperative outcome assessment (2, 3).

Traditionally, the establishment of the FHP has relied primarily on two-dimensional lateral cephalometric radiographs, in which the plane is approximated by manually identifying superimposed bilateral anatomical landmarks. It is defined as the horizontal plane passing through the orbitale points (Or), corresponding to the lowest points of the infraorbital margins, and the porion points (Po). However, inherent limitations of two-dimensional imaging—including projection superimposition, image magnification, head posture deviation (4), and the subjectivity associated with landmark identification (5)—often compromise the reproducibility of FHP construction. These limitations are particularly pronounced in patients with facial asymmetry or complex deformities, where such errors may be further amplified, potentially affecting the objectivity and reliability of diagnostic and therapeutic decision-making (6).

In recent years, with rapid advances in medical imaging technology, cone-beam computed tomography (CBCT) has become an important modality for craniofacial structural assessment owing to its high spatial resolution, relatively low radiation dose, and true three-dimensional imaging capability (7). CBCT provides isotropic volumetric data that support multiplanar reconstruction and 3D model generation, enabling accurate and spatially precise identification of anatomical landmarks. On this basis, FHPs constructed using specialized 3D reconstruction software, such as Mimics (8, 9) and Geomagic (10), can faithfully reproduce the spatial geometry of the craniofacial skeleton while effectively overcoming projection-related errors and posture-related interference inherent to two-dimensional imaging. Consequently, CBCT-based approaches substantially enhance the anatomical reliability and operational reproducibility of FHP establishment.

Several researchers, including Pittayapat P (11) and Gauthier Dot (12), have proposed novel CBCT-based methods for defining the FHP, in which the plane is established by identifying the orbitale (Or) and the internal auditory foramen (IAF), with the midpoint between the bilateral IAFs and the orbitale midpoint (Orm) used to construct the reference plane. This 3D landmarking strategy replaces the conventional 2D Or–Po definition. Although these studies have systematically evaluated the reproducibility of CBCT-based FHP construction, including repeatability and agreement, there is still a lack of standardized and quantitatively validated approaches for three-dimensional FHP construction. Moreover, although the superiority of CBCT over conventional two-dimensional cephalometric methods has been well established, there is still a need for a unified framework to interpret the reproducibility of Frankfort horizontal plane construction in clinically meaningful terms.

Therefore, further methodological investigation is warranted to establish a reliable and reproducible three-dimensional framework for FHP construction, with quantitative validation and clinically interpretable metrics. The purpose of the present study was to establish the Frankfort horizontal plane using CBCT-based 3D reconstruction techniques, systematically evaluate its repeatability and agreement, and evaluate the reproducibility errors associated with lateral cephalometric radiographs in FHP construction. By establishing a quantitatively validated and reproducible three-dimensional workflow for Frankfort horizontal plane construction, this study aims to provide a more reliable and clinically applicable reference framework for craniofacial diagnosis and treatment planning. These findings are expected to have important theoretical and practical implications for advancing precision orthodontics and orthognathic surgery, as well as for enhancing the objectivity and scientific rigor of clinical practice. Moreover, CBCT-based 3D reconstruction may serve as a source of high-quality, standardized annotation data for future artificial intelligence models (13, 14), thereby facilitating the automation and precision of deep learning algorithms in clinical applications and laying a solid anatomical foundation for intelligent clinical decision-support systems.

## 2 Material and methods

### 2.1 Study subjects

This study was reviewed and approved by the Ethics Committee of the Eighth Affiliated Hospital of Southern Medical University (Approval No. KYLS20240910). All participants were fully informed of the study protocol and provided written informed consent prior to enrollment. A total of 27 patients(mean age 28.6 ± 7.2 years; 15 females, 12 males) who presented to the Department of Oral and Maxillofacial Surgery or the Department of Orthodontics at our institution for the treatment of malocclusion between January 2024 and June 2025 were consecutively enrolled.

#### 2.1.1 Inclusion criteria

Participants were eligible for inclusion if they met the following criteria:

1. age between 18 and 50 years;
2. a definitive diagnosis of facial deformities confirmed by clinical examination and imaging findings, including but not limited to developmental jaw asymmetry, mandibular prognathism or retrognathism, maxillary hypoplasia, and laterognathic deformities;
3. availability of both head CBCT scans and standard lateral cephalometric radiographs obtained for diagnostic or treatment-planning purposes.

#### 2.1.2 Exclusion criteria

Patients were excluded if any of the following conditions were present:

1. a history of craniofacial trauma or previous orthognathic, reconstructive, or cosmetic surgery, to avoid interference with landmark identification due to altered skeletal anatomy;
2. inadequate image quality, including severe metal artifacts, motion artifacts, or incomplete scanning coverage that prevented clear visualization of key anatomical landmarks;
3. systemic diseases, syndromic conditions, or neoplastic disorders affecting craniofacial skeletal structures.

All CBCT and lateral cephalometric data from eligible patients were anonymized prior to analysis to ensure patient confidentiality.

### 2.2 Data acquisition and processing

Large–field-of-view cone-beam computed tomography (CBCT) scans were obtained using a cone-beam CT scanner (NewTom VG Volumetric Scanner, Aperio, Italy). The scanning range extended from the cranial vertex to the menton. All imaging data were stored in Digital Imaging and Communications in Medicine (DICOM) format. CBCT parameters: voxel size 0.3 mm, FOV 18 × 24 cm, 80 kV, 5 mA. Patients were positioned with standardized head posture and maximum intercuspation. CBCT and lateral radiographs were obtained within 2 weeks.

The CBCT DICOM datasets were imported into Mimics Medical 21.0 (Materialise, Leuven, Belgium) for image processing. In the segmentation module, a threshold of 700–3071 HU was selected to isolate hard tissue. 3D reconstruction was then performed to generate digital cranial models, which were subsequently exported and saved in stereolithography (STL) format for further analysis.

For craniofacial hard-tissue segmentation, a threshold range of 700–3071 HU was applied to all CBCT datasets. Previous studies have demonstrated that segmentation threshold selection is highly dependent on imaging modality, scanner characteristics, anatomical structures, and reconstruction objectives (15, 16), and that no universally accepted threshold exists for craniofacial reconstruction (17). The threshold range of 700–3071 HU was selected based on preliminary pilot testing performed before formal data collection, because it provided consistent visualization of craniofacial bony structures while minimizing inclusion of non-osseous tissues and image noise. The same threshold range was subsequently applied to all datasets to ensure methodological consistency throughout the study. Visual inspection of five randomly selected scans was used only as a quality-control procedure to confirm preservation of anatomical boundaries after segmentation.

### 2.3 Generation of the Frankfort horizontal plane

#### 2.3.1 Landmark identification and annotation

The cranial STL files were imported into the medical three-dimensional reverse engineering software Geomagic Studio 2014 (3D Systems, USA). Two senior orthodontists, each with more than 5 years of clinical experience, independently performed landmark identification and annotation without mutual communication. All procedures were conducted on the same calibrated monitor using the three-dimensional coordinate system provided by the Mimics software.

The key anatomical landmarks identified and annotated were as follows:

1. Orbitale (Or):
  Defined on the 3D model as the lowest point on the infraorbital margin on each side. Landmark positioning was confirmed through repeated observations in multiple views, including sagittal, coronal, axial planes, and the 3D rendered model, to ensure reproducibility.
2. Internal acoustic foramen (IAF):
  Defined on the 3D model as the innermost point of the internal acoustic foramen on each side. Multiplanar visualization was similarly employed to verify precise localization.

Each orthodontist independently completed the initial annotation of all four bilateral landmarks for each patient, including the left and right Or points and the left and right IAF points.

#### 2.3.2 Construction of the three-dimensional Frankfort horizontal plane

In Geomagic Studio 2014, the midpoint between the left and right Or points was calculated to define the orbitale midpoint (Orm). Together with the left and right IAF points, a plane passing through these three points was generated. This plane was defined as the 3D Frankfort horizontal plane (FHP) used for subsequent analysis. The normal vector of the plane was extracted and used as the directional reference of the FHP in 3D space (Supplementary Figure 1).

### 2.4 Reproducibility assessment

To comprehensively evaluate the reliability of Frankfort horizontal plane (FHP) construction based on CBCT 3D reconstruction, reproducibility was quantitatively assessed at both the landmark and plane levels, including intra-observer and inter-observer reproducibility. The results were further compared with those obtained using the conventional lateral cephalometric radiographic method.

Prior to the commencement of formal data collection, both observers completed a standardized calibration protocol, which included training on landmark definition, 3D visualization, and planar construction. The study selected five samples for a pilot experiment to assess preliminary inter-group consistency; points of disagreement were discussed and standardized criteria were established to minimize inter-observer errors. Five pilot samples were not included in final analysis. Observers were blinded to patient identity and prior measurements; landmark order was randomized.

#### 2.4.1 Landmark positional error assessment

All 3D models were analyzed using the default coordinate system in Geomagic Studio 2014. For one patient, annotations were completed in the same file to ensure consistent coordinate axes.

Intra-observer reproducibility: One week after completion of the initial landmark identification, the same orthodontist performed a second round of anonymized landmark annotation for the bilateral internal acoustic foramen (IAF) and orbitale midpoint (Orm) in all patients, without access to the initial results. Consistency of the 3D coordinates was assessed, and the three-dimensional Euclidean distance (mm) between corresponding landmarks identified in the two sessions by the same observer was calculated. This distance was defined as the intra-observer landmark positional error.

Inter-observer reproducibility: The three-dimensional Euclidean distance (mm) between corresponding landmarks identified by the two orthodontists in each annotation session was directly calculated. This distance was defined as the inter-observer landmark positional error.

#### 2.4.2 Plane angular error assessment

Intra-observer reproducibility: Based on the landmarks identified in the two annotation sessions by each observer, two three-dimensional FHPs were generated. The angle (°) between the normal vectors of these two planes was calculated and defined as the intra-observer plane angular error.

Inter-observer reproducibility: Based on the landmarks identified during the initial annotation session by the two observers, two three-dimensional FHPs were constructed. The angle (°) between the normal vectors of these two planes was calculated and defined as the inter-observer plane angular error.

#### 2.4.3 Assessment of reproducibility errors in CBCT and lateral cephalometric methods

On the lateral cephalometric radiographs of each patient, bilateral orbitale and porion landmarks were manually identified by both observers using Adobe Photoshop 2024 (Adobe Inc., USA). A two-dimensional Frankfort horizontal plane was constructed by connecting the identified landmarks (Supplementary Figure 2).

For intra-observer reproducibility assessment, each observer repeated landmark identification after a one-week interval. The orientation angle of each FH line was measured relative to the image coordinate system, and the intra-observer FH-line angular error was calculated as the absolute difference between the two repeated orientation angles. For inter-observer reproducibility assessment, the FH-line angular error was calculated as the absolute difference between the FH-line orientation angles independently constructed by the two observers.

For both modalities, reproducibility was quantified using observer-dependent orientation errors expressed in degrees. Although the geometric entities differed (a three-dimensional plane in CBCT and a two-dimensional line in lateral cephalometry), both metrics represented variability in the orientation of the Frankfort horizontal reference resulting from repeated landmark identification and reference construction. Therefore, the resulting angular errors were interpreted as workflow-specific indicators of reproducibility rather than measures of geometric equivalence between a three-dimensional plane and a two-dimensional line.

#### 2.4.4 Statistical analysis

All landmark positional errors and plane angular errors were expressed as mean ± standard deviation (SD). Statistical analyses were performed using SPSS software (version 26.0; IBM Corp., Armonk, NY, USA). Intra-observer reproducibility of CBCT-based 3D landmark identification was assessed using intra-class correlation coefficients (ICCs). Descriptive statistical analyses were conducted for both landmark positional errors and plane angular errors. Bland–Altman analysis was applied to evaluate the limits of agreement for the CBCT-based method.

To evaluate differences in reproducibility errors generated by the CBCT-based and lateral cephalometric reference-construction workflows, paired t tests were performed. A p value of less than 0.05 was considered statistically significant. Sample size was determined *a priori* for ICC-based reliability analysis. Parameters: expected ICC = 0.90, minimum acceptable ICC = 0.75, *α* = 0.05, power = 0.80, 2 observers, 2 repetitions. Calculations were performed using PASS 15.0 (NCSS, LLC, USA), yielding a required sample size of 24. Twenty-seven patients were enrolled, satisfying the requirement. ICC values with 95% CIs are reported.

## 3 Results

All procedures were successfully executed on all digital cranial models and lateral cephalometric radiographs using both methods. The intra-observer reproducibility of landmark coordinates was evaluated using the two-way mixed-effects intraclass correlation coefficient (ICC) with absolute agreement, single-measurement model (Supplementary Table 1). The results demonstrated that all landmarks exhibited ICC values greater than 0.90 in all three spatial coordinate directions, indicating excellent intra-observer consistency and confirming the high reproducibility of the CBCT-based landmarking method.

Three-dimensional landmark positional errors (Supplementary Table 2) and FHP plane angular errors (Supplementary Table 3) were subsequently calculated for the CBCT-based reconstructions. To assess agreement, Bland–Altman analysis was conducted comparing the CBCT-identified landmarks and FHPs, both for repeated annotations by the same observer and between the two observers (Supplementary Table 4). The Bland–Altman plots indicated that the mean biases were all close to zero, indicating no systematic error. The 95% limits of agreement were narrow, with most values falling within the thresholds of ±1° for angles and ±2 mm for positions.

Reproducibility errors generated by the CBCT-based and lateral cephalometric workflows were subsequently evaluated. The normality of data was verified using the Shapiro–Wilk test (all *P* > 0.05, confirming normal distribution). Paired t tests were performed to evaluate intra-observer and inter-observer reproducibility between the two methods, and effect sizes were reported using Cohen's d, with |d| ≥ 0.8 interpreted as a large effect (Supplementary Table 5). The reproducibility errors generated by the two workflows differed significantly (*p* < 0.001). Specifically, the mean reproducibility error for the CBCT group (0.87) was markedly lower than that of the lateral cephalometric group (1.72), indicating lower reproducibility errors in the CBCT-based workflow.

## 4 Discussion

In this study, we systematically investigated the reproducibility of FHP construction using CBCT-based 3D reconstruction and evaluated reproducibility errors generated by CBCT and lateral cephalometric workflows. CBCT-based 3D methods demonstrated lower reproducibility errors than the lateral cephalometric workflow under the conditions of the present study.

Our findings indicate that for repeated annotations by the same observer, the mean landmark positional error was less than 1.2 mm and the plane angular error was less than 0.92°. For inter-observer comparisons, the mean landmark positional error was less than 1.35 mm and the plane angular error was less than 0.94°. These results are consistent with previously reported reproducibility data (18, 19), confirming that the CBCT-based method exhibits excellent reliability. The observed lower reproducibility errors may be attributed to several factors. First, the isotropic three-dimensional volumetric data provided by CBCT allows operators to simultaneously observe and verify anatomical landmarks across sagittal, coronal, and axial planes (20), enabling a transition from “approximate estimation” to “precise localization” (21, 22). For example, when identifying the porion, the operator can precisely locate the innermost point of the bony internal auditory canal in the axial plane while simultaneously confirming its position in the coronal and sagittal planes, minimizing potential errors from single-plane assessment (23). However, the presence of relatively large maximum errors warrants caution. These maximum values suggest that in cases with complex anatomy or severe asymmetry, the reproducibility of landmark identification may decrease, potentially affecting the accuracy of surgical planning and outcome evaluation. Clinicians should be aware of this variability and consider cross-verifying key landmarks in such cases.

Mimics software enables reconstruction of a precise 1:1 3D craniofacial model from CBCT data, providing a realistic, distortion-free spatial basis for subsequent analysis (24). Finally, in Geomagic Studio, a plane is directly constructed using the orbitale midpoint (Orm) and bilateral internal auditory foramen (IAF) points based on explicit geometric definitions. This approach is logically straightforward and rigorously defined, avoiding additional variability that may be introduced by complex algorithms, and ensuring transparency and interpretability in plane generation. Moreover, minor unilateral landmark identification deviations can be effectively accommodated, as the use of midpoints balances left–right asymmetry, enhancing the robustness and clinical applicability of the resulting FHP.

In sharp contrast to the high reproducibility observed with CBCT, lateral cephalometric radiographs exhibited significantly larger plane angular errors in both intra-observer and inter-observer comparisons. Specifically, for repeated annotations by the same observer, CBCT reduced the mean angular error by 0.829° compared to lateral cephalograms; for inter-observer comparisons, CBCT reduced the mean error by 0.871°. These findings directly highlight the inherent limitations of two-dimensional imaging when reconstructing 3D anatomical structures. The main sources of error in lateral cephalometric FHP construction include:

- Projection superimposition and landmark ambiguity: On two-dimensional lateral cephalograms, bilateral orbitale and porion points overlap, making it impossible to distinguish left from right. Operators must estimate positions based on grayscale and contour, or subjectively select a “representative” point. In patients with facial asymmetry, inherent left–right positional differences are forcibly collapsed into a single plane, introducing significant systematic errors (25). Durão et al. (26) reported that among 17 commonly used cephalometric landmarks, orbitale exhibited the lowest reliability with an SD of 4.41 mm.
- Head posture variations: Minor tilts or rotations during lateral radiograph acquisition can markedly alter the projected landmark positions, compromising FHP reproducibility (27, 28). In contrast, CBCT-based 3D reconstructions allow spatial alignment and standardization, eliminating positional variability.
- Image magnification and distortion: Geometric projection in lateral cephalograms can result in image magnification and edge distortion. Balachandran et al. (29) reported an inherent 33% magnification in digital lateral cephalograms of the skull. Consequently, FHPs derived from lateral cephalograms are less accurate and reliable than those from CBCT, limiting their utility in precision-demanding modern orthognathic surgery and orthodontics.

CBCT-based 3D reconstruction provides a highly reliable and reproducible craniofacial reference plane for patients with facial deformities, particularly those with asymmetrical malformations. Feng et al. (30) developed a standardized and dependable temporomandibular joint (TMJ) assessment reference system based on the FHP, enabling comprehensive evaluation of TMJ positional and morphological changes during treatment follow-up. This advancement is of critical importance for promoting precision in clinical diagnosis and therapy.

In clinical practice, using a highly reproducible three-dimensional FHP as a reference plane can substantially enhance the reproducibility of cephalometric measurements (31–33), surgical simulation (34), surgical guide design, and postoperative outcome assessment (35). For instance, when planning bimaxillary orthognathic surgery for patients with laterognathic deformities, an FHP that accurately reflects the 3D spatial relationships of the cranial base is fundamental for correctly analyzing deformity mechanisms and calculating the required skeletal movements. Lee KJC (36) compared the reproducibility of digital workflows for bimaxillary orthognathic surgery and noted considerable linear discrepancies between simulated and actual surgical outcomes, highlighting the need to further improve the precision of digital reconstructions to minimize surgical errors. The standardized workflow established in the present study demonstrates broad applicability and shows higher reproducibility.

The advantages of CBCT over conventional two-dimensional radiography in craniofacial imaging have been widely reported in previous studies (21, 22). The present study does not aim to reconfirm this well-established principle, but to provide specific and quantitative evidence regarding the reproducibility of the FHP. The novelty of this study lies in its detailed evaluation of 3D coordinate-based reproducibility (X, Y, Z axes), strict standardization of the 3D model orientation, and comparison against clinical acceptability thresholds using Bland–Altman analysis and effect sizes. These findings support that CBCT-based 3D reconstruction demonstrates higher reproducibility in FHP construction, providing methodological reference for clinical use.

This study has several limitations. First, the sample size was relatively small and comprised patients from a single medical center, which may limit the generalizability of the findings to more diverse or complex deformities, such as severe craniosynostosis or syndromic malformations. Future studies should consider multi-center collaboration to increase sample size and stratify subgroups by deformity type and severity, providing a more comprehensive assessment of method stability across different populations. Second, this study primarily focused on FHP reproducibility; future research could extend to evaluating the reproducibility of other measurements derived from this reliable reference plane. Third, the current workflow relies on specialized software platforms such as Mimics and Geomagic, and manual landmark annotation requires experienced clinicians, making the process time-consuming and operator-dependent. In addition, a fixed segmentation threshold range of 700–3071 HU was applied to all CBCT datasets to ensure consistency of the reconstruction workflow. Although this threshold range was selected based on preliminary testing and quality-control procedures, different threshold values may influence the reconstructed anatomy and could potentially affect reproducibility measurements. Future studies should investigate the impact of different segmentation thresholds on FHP reconstruction reproducibility through dedicated sensitivity analyses. These factors may limit rapid adoption in routine clinical practice and should be addressed in future automated and standardized workflows.

However, recent advances in artificial intelligence, including deep learning, have demonstrated substantial progress in CBCT automatic segmentation (37, 38), image recognition (39–41), and mandibular growth prediction (42). The precisely annotated dataset provided in this study offers a high-quality “gold standard” for training automated algorithms. Developing AI models capable of automatically identifying critical landmarks, such as orbitale and porion, represents a key objective for future work and is essential for achieving both precision and efficiency in clinical applications.

## Data Availability

The raw data supporting the conclusions of this article will be made available by the corresponding author upon reasonable request.
