# Supplementary material for: Study on the reproducibility of the Frankfort horizontal plane based on CBCT 3D reconstruction and comparative analysis with lateral radiographs
Source: Front Surg. 2026 Jul 16;13:1786145. doi: 10.3389/fsurg.2026.1786145 (PMC13420836; doi:10.3389/fsurg.2026.1786145)
Supplement: Supplementary file 1 [file Datasheet1.docx]

Supplementary Material


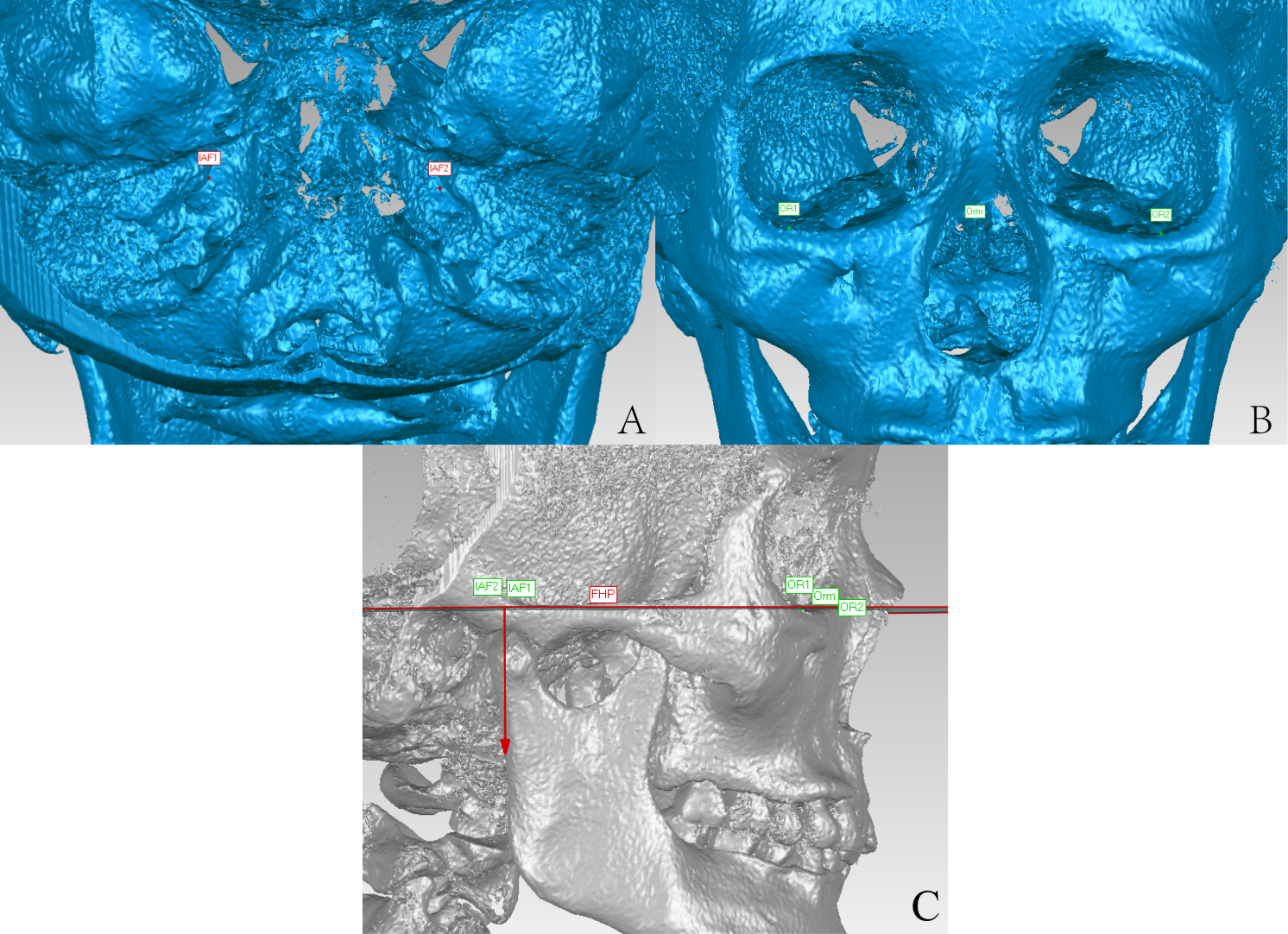


**Figure 1** Construction of the three-dimensional Frankfort horizontal plane (A: bilateral internal auditory canal point annotation; B: bilateral infraorbital point and midpoint annotation; C: construction of the Frankfort horizontal plane)


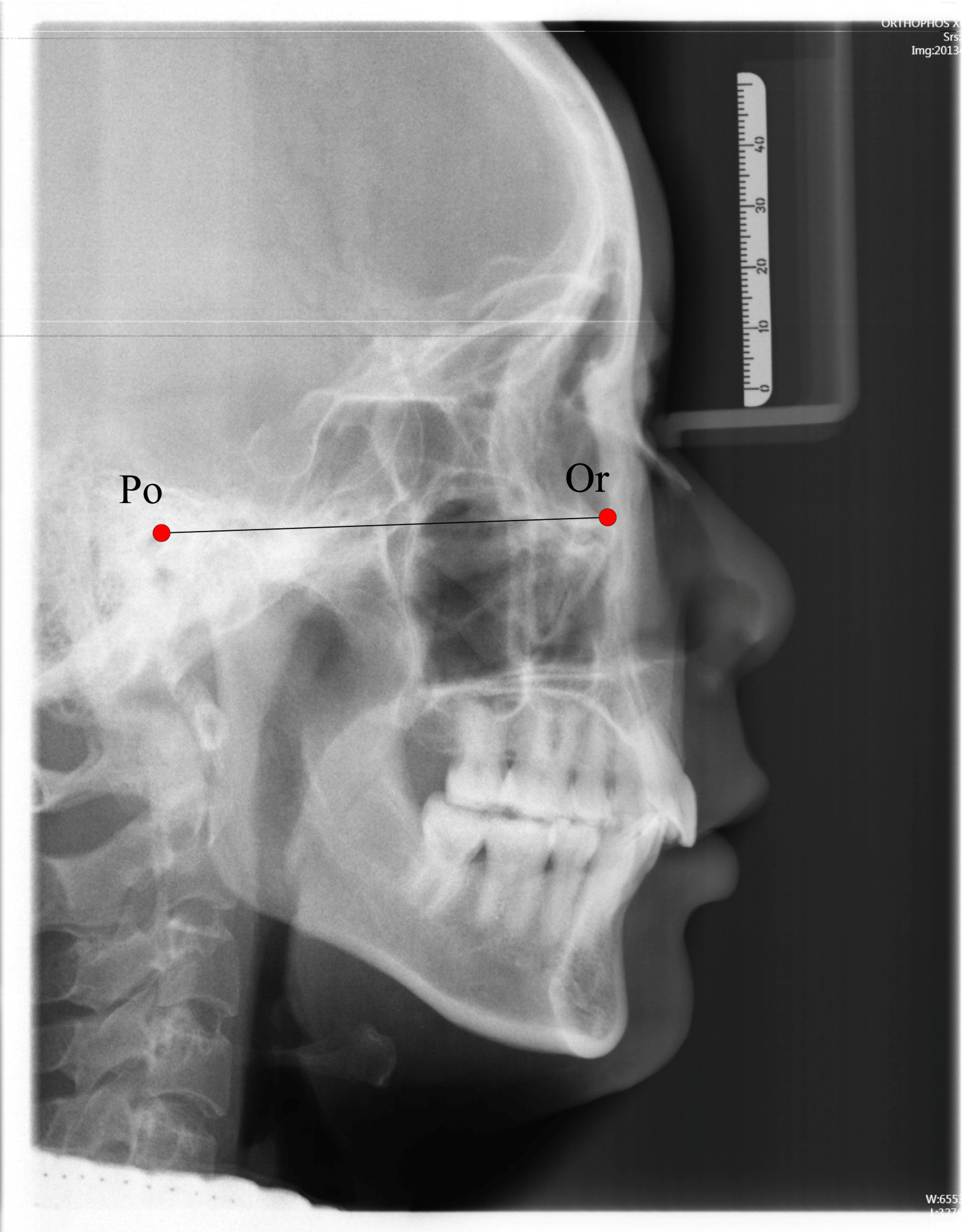


**Figure 2** Construction of the Frankfort horizontal plane on lateral radiographs

**Table 1**  The reproducibility of CBCT annotation in three-dimensional reconstruction by the same doctor

|  | **X** | **Y** | **Z** |
| --- | --- | --- | --- |
| ICCs | 0.943 | 0.967 | 0.997 |
| 95% CI | 0.903 ~ 0.966 | 0.943 ~ 0.980 | 0.994 ~ 0.998 |

**Table 2**  Point position errors of the Frankfort horizontal plane labeled by CBCT (mm)

| **Groups** | **Max** | **Mean** | **Standard deviation** | **95% CI of Mean** |
| --- | --- | --- | --- | --- |
| A1-A2 | 3.92 | 1.194 | 0.766 | 0.905 ~ 1.483 |
| B1-B2 | 3.139 | 1.085 | 0.752 | 0.802 ~ 1.368 |
| A1-B1 | 3.746 | 1.344 | 0.976 | 0.976 ~ 1.712 |
| A2-B2 | 3.47 | 1.309 | 0.848 | 0.989 ~ 1.629 |

A1: First annotation by Doctor A; A2: Second annotation by Doctor A; B1: First annotation by Doctor B; B2: Second annotation by Doctor B;

**Table 3** Angle errors of the Frankfort horizontal plane labeled by CBCT (°)

| **Groups** | **Max** | **Mean** | **Standard deviation** | **95% CI of Mean** |
| --- | --- | --- | --- | --- |
| A1-A2 | 1.834 | 0.918 | 0.423 | 0.758 ~ 1.078 |
| B1-B2 | 1.923 | 0.896 | 0.435 | 0.732 ~ 1.060 |
| A1-B1 | 2.258 | 0.939 | 0.534 | 0.738 ~ 1.140 |
| A2-B2 | 1.701 | 0.713 | 0.463 | 0.538 ~ 0.888 |

A1: First annotation by Doctor A; A2: Second annotation by Doctor A; B1: First annotation by Doctor B; B2: Second annotation by Doctor B;

**Table 4** Bland-Altman analysis of two annotations by the same doctor and annotations between two doctors


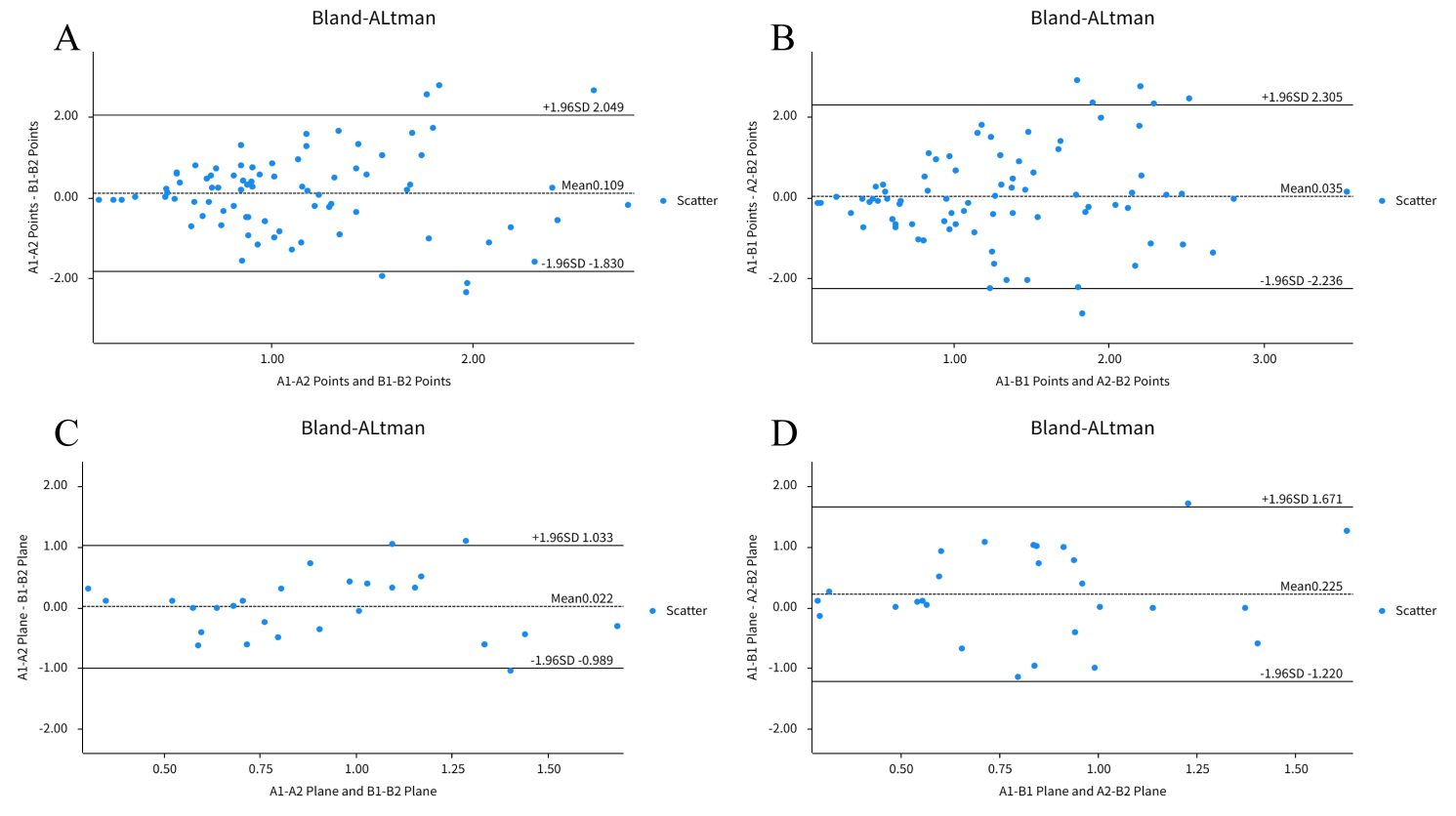


(A) Intra-observer agreement for point position; (B) Inter-observer agreement for point position; (C) Intra-observer agreement for plane angle; (D) Inter-observer agreement for plane angle.

The dashed line represents the mean bias, and the solid lines represent the 95% limits of agreement (LoA). The analysis confirms good agreement, with most values falling within clinically acceptable thresholds.

**Table 5** Comparison of reproducibility between CBCT and lateral radiography (°)

| **Groups** | **Mean** | **Standard deviation** | **95% CI of Mean** | **Mean Difference** | ***t*** | ***p*** | ***Cohen’s d*** |
| --- | --- | --- | --- | --- | --- | --- | --- |
| CBCT | 0.87 | 0.47 | 0.69 ~ 1.05 | -0.85 | -11.758 | 0.000**** | 2.26 |
| Lateral radiography | 1.72 | 0.62 | 1.48 ~ 1.96 |  |  |  |  |

*****P＜0.001*
